# Carbon dioxide and particulate emissions from the 2013 Tasmanian firestorm: implications for Australian carbon accounting

**DOI:** 10.1186/s13021-022-00207-9

**Published:** 2022-05-26

**Authors:** Mercy N. Ndalila, Grant J. Williamson, David M. J. S. Bowman

**Affiliations:** School of Natural Sciences, University of Tasmania, Hobart, TAS 7001 Australia

**Keywords:** Wildfire, Emission, Carbon, Particulate, Smoke, PyroCb, *Eucalyptus*, GFED, FullCAM

## Abstract

**Background:**

Uncontrolled wildfires in Australian temperate *Eucalyptus* forests produce significant smoke emissions, particularly carbon dioxide (CO_2_) and particulates. Emissions from fires in these ecosystems, however, have received less research attention than the fires in North American conifer forests or frequently burned Australian tropical savannas. Here, we use the 2013 Forcett–Dunalley fire that caused the first recorded pyrocumulonimbus event in Tasmania, to understand CO_2_ and particulate matter (PM_2.5_) emissions from a severe *Eucalyptus* forest fire. We investigate the spatial patterns of the two emissions using a fine scale mapping of vegetation and fire severity (50 m resolution), and utilising available emission factors suitable for Australian vegetation types. We compare the results with coarse-scale (28 km resolution) emissions estimates from Global Fire Emissions Database (GFED) to determine the reliability of the global model in emissions estimation.

**Results:**

The fine scale inventory yielded total CO_2_ emission of 1.125 ± 0.232 Tg and PM_2.5_ emission of 0.022 ± 0.006 Tg, representing a loss of 56 t CO_2_ ha^−1^ and 1 t PM_2.5_ ha^−1^. The CO_2_ emissions were comparable to GFED estimates, but GFED PM_2.5_ estimates were lower by a factor of three. This study highlights the reliability of GFED for CO_2_ but not PM_2.5_ for estimating emissions from *Eucalyptus* forest fires. Our fine scale and GFED estimates showed that the Forcett–Dunalley fire produced 30% of 2013 fire carbon emissions in Tasmania, and 26–36% of mean annual fire emissions for the State, representing a significant single source of emissions.

**Conclusions:**

Our analyses highlight the need for improved PM_2.5_ emission factors specific to Australian vegetation, and better characterisation of fuel loads, particularly coarse fuel loads, to quantify wildfire particulate and greenhouse gas emissions more accurately. Current Australian carbon accountancy approach of excluding large wildfires from final GHG accounts likely exaggerates Tasmania’s claim to carbon neutrality; we therefore recommend that planned and unplanned emissions are included in the final national and state greenhouse gas accounting to international conventions. Advancing these issues is important given the trajectory of more frequent large fires driven by anthropogenic climate change.

**Supplementary Information:**

The online version contains supplementary material available at 10.1186/s13021-022-00207-9.

## Background

Fire plays an important role in the functioning of many terrestrial ecosystems globally and affects climate via the release of greenhouse gases (GHGs) and aerosols in smoke. Emerging evidence suggests that climate change is causing worsening fire weather, longer fire seasons and more intense wildfires globally [1]. Frequent and intense fires have the potential to release enormous quantities of greenhouse gases, thereby exacerbating climate change in a positive feedback process. Carbon dioxide (CO_2_) contributes the largest proportion of total wildfire smoke emissions (90% of carbon emissions) and is therefore an important driver of radiative forcing [2]. CO_2_ is assimilated by plants in subsequent growing seasons post-fire; however, frequent fires and changing climate may limit the ability of ecosystems to recover from the fires, resulting in net positive CO_2_ emissions [3]. Another important product of wildfire combustion is particulate emission which accounts for < 5% of total carbon emissions [4]. Smoke particles affect climate in complex and poorly understood ways causing both short term regional climate cooling due to regional haze formation [5], somewhat analogous to volcanic eruptions [6], and also atmospheric warming, affecting precipitation patterns [7]. Particulates (especially PM_2.5_, the fraction of particles with a diameter < 2.5 µm) have an important and demonstrable harmful effects on human health, including worsened respiratory symptoms, exacerbation of respiratory and cardiovascular diseases, and premature mortality from cardiovascular complications [8].

These issues are well illustrated by fire activity in Australian temperate forests that have experienced increased fire danger due to extreme fire weather conditions, with resultant lengthening of fire seasons earlier into spring months, associated with climate change [9]. Further, the recent 2019–2020 Black Summer fires in south-eastern Australia are historically unprecedented and most likely exacerbated by climate change [10–12]. Analyses involving remote sensing of atmospheric chemistry suggest that the Black Summer fires emitted 715 Tg of CO_2_ [13], in broad agreement with a bootstrapped emissions estimate of c 670 Tg [3]. It is estimate that 0.3–1.1 Tg of smoke particles were injected into the stratosphere by these fires [14]. Associated particulate pollution from the 2019–2020 fires is estimated to have caused premature death of 429 people and caused nearly 2 billion Australian dollars in health costs [15]. The emissions for the 2019–2020 season are estimated to be 80 times higher than the average fire season apparent in the satellite record [16], highlighting the importance of understanding the impacts of wildfires on GHG emissions.

Despite their capacity to pollute the atmosphere, there are surprisingly few studies of carbon and particulate emission from individual Australian fires. Savanna fires in northern Australia have received the greatest attention, motivated by interest in landscape carbon abatement programs, e.g., [17–19]. In temperate *Eucalyptus* forests, the majority of the studies are based on emissions from prescribed fires, e.g., [20–22] with a few exceptions involving laboratory measurements, e.g., [23] or wildfires, e.g., [24, 25]. Particulate emissions from Australian fires still remain largely unexplored, with one study conducted from prescribed fires in south-eastern Australia [26] and a second study on Black Summer fires [14]. A consequence of this limited inquiry is that global analyses often extrapolate these few studies to the entire Australian continent, or use gaseous and particulate emission coefficients from other biomes globally, especially North America, or both. For instance, a frequent source of emissions data is the Global Fire Emissions Database (GFED), which is the most widely used global emissions inventory and has also been critical in assessing the global and regional burden of mortality due to PM_2.5_ pollution from landscape fires [27, 28].

Accurate estimation of carbon emissions is important for a complete understanding of regional and national carbon accounts. Emissions from Australian wildfires are accounted for in the national GHG accounting to the Intergovernmental Panel on Climate Change (IPCC); however, very large fires are attributed as natural disturbances, so they are excluded in the final fire-related emissions estimation [29]. This approach likely affects the claim of ‘carbon neutrality’ by the state of Tasmania given a spate of large wildfires that have burned around 25% of the island since 1990.

Accurate assessments of particulate emissions are essential for quantifying the exposure of populations to smoke pollution, and in assessing the trade-offs in health impacts from prescribed fires and wildfires [30]. Beyond substantial health costs, particulates have, as aforementioned, a demonstrable harmful impact on human health with regard to cardiovascular and respiratory complications [27]. Particulate emission estimation is also important from a climate perspective because of their influence on haze and cloud dynamics that affects atmospheric chemistry and radiative balance at regional and hemispherical scales [31].

The January 2013 Forcett–Dunalley fire presents an ideal model system to understand smoke emissions from a single, intense fire in a southeast Australian temperate *Eucalyptus* forest. This fire is notable because it generated a pyrocumulonimbus (PyroCb)—a fire-induced thunderstorm that almost destroyed a small town [32], with the fire burning 25,950 ha of natural *Eucalyptus* forests, *Eucalyptus* plantations and agricultural lands. Approximately 55% of the area burnt as high-very high severity, under the influence of extreme weather and dry fuels in the landscape, coupled with a conducive undulating terrain that amplified the fire intensity, estimated to reach c. 68,000 kW m^−1^ [33]. The PyroCb from the fire was the first record for the island state, although it is becoming increasingly common across eastern Australia and in North America, likely due to climate change [14].

In this study, we test the hypothesis that CO_2_ and PM_2.5_ emissions from a single intense wildfire that were estimated from an existing geographically coarse-scale global model is closely correlated with estimates from a purpose-built local model using spatially high-resolution inputs. We then explore how global and local scale wildfire emission estimates can improve regional and national carbon accounting approaches and thereby shape the understanding of carbon ‘costs’ of wildfires. Building on prior analyses of the fire [33] and the bootstrapped emissions analysis of Bowman et al. [3], we: (1) use fine-scale mapping of vegetation and fire severity to map the spatial distribution of CO_2_ and fine particulate matter (PM_2.5_) emissions from the fire using the original model by Seiler and Crutzen [34]; (2) compare the spatial distribution and total emissions of the two pollutants between the basic model and the global GFED model to determine the effect of geographical resolution of fire severity and vegetation mapping on emissions estimation; (3) compare daily emissions estimates between the two inventories during the days of concurrently recorded fire activity (3–18 January 2013); and (4) contextualise the Forcett–Dunalley emissions and determine by how much the emissions contribute to overall wildfire and GHG emissions in Tasmania. This study is limited to estimation of CO_2_; estimation of additional gaseous species such as methane and nitrous oxides could in future be scaled beyond CO_2_ and expressed as CO_2_-equivalent emissions.

## Methods

### Study area

**Fig. 1:**
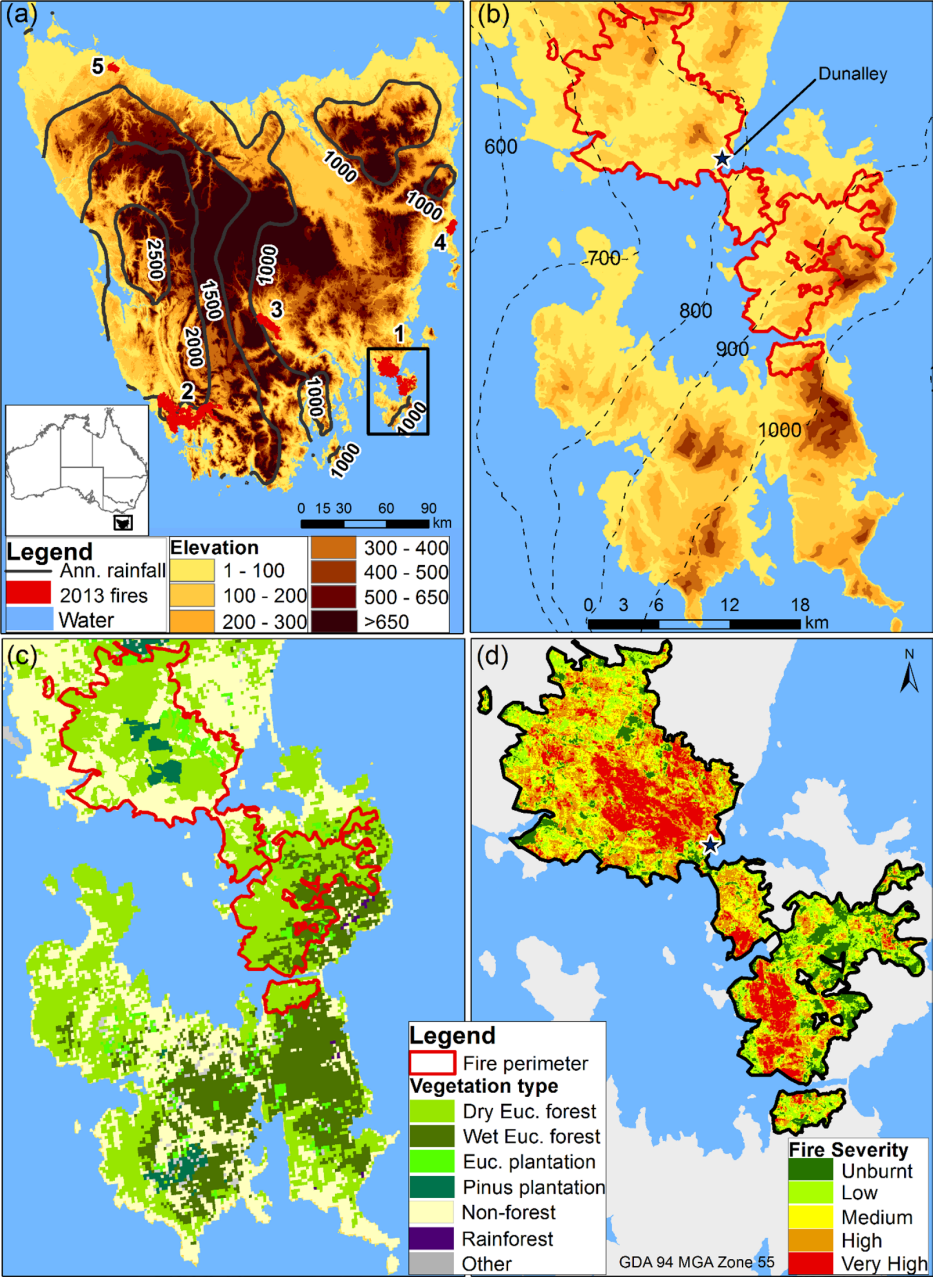
Location of the Forcett–Dunalley fireground in SE Tasmania: **a** Annual rainfall (in mm) and elevation (in m) across Tasmania and the location of major fires in the 2013 fire season including Forcett–Dunalley (1). **b** Elevation and mean annual rainfall across the Forestier and Tasman Peninsulas, derived from Worldclim dataset [35]. The location of Dunalley township is indicated on the map. **c** Dominant vegetation in the Forestier and Tasman Peninsulas based on TASVEG 3.0, an integrated vegetation map of Tasmania. **d** Fire severity patterns within the fireground. Adapted from Ndalila et al. [33]

The Forcett–Dunalley fireground has a cool temperate climate with annual rainfall of 700–1000 mm, mean daily temperature of 17 °C in summer and 9 °C in winter, and elevation rising from sea-level to 600 m above sea level (Fig. 1b). Native *Eucalyptus* forests, and *Pinus* and *Eucalyptus* plantations are found within the area, with the dry *Eucalyptus* forest as the most dominant vegetation type (Fig. 1c). The fire occurred from 3 to 18 January 2013 on the Forestier and Tasman Peninsulas in the south-east of Tasmania, the southernmost island state of Australia (Fig. 1a). The fire was ignited possibly from a smouldering stump from an unextinguished campfire. The fire burnt under varying fire weather conditions, topography and fuel characteristics leading to spatial variability in fire severity within the fireground (Fig. 1d). By the time of containment, the fire had burnt approximately 20,200 ha of the 25,950 ha fireground, mostly affecting native vegetation and rural lands (Fig. 1c). A detailed description of the fire and associated broader environmental conditions have been provided in Ndalila et al. [33].

### Data preparation

Emission factors (EFs) in our study represent the total mass of a series of gaseous or particulate species emitted per mass of dry fuel burnt. In order to calculate total emissions from biomass burning over a defined area, emission factors are multiplied by the mass of fuel consumed, in a relationship defined by Eq. 1 [34]. The equation incorporates emissions factors (EFs) for the emitted gases and particulates, in addition to the standard estimates of area burnt, fuel loads and the fraction of fuel consumed. A grid covering the extent of the fire perimeter, with 50 m-resolution grid cells, was used for the emissions analysis.

1 $$\mathrm{E}i =\mathrm{ A }(x) *\mathrm{ FL }(x) *\mathrm{ CC }*\mathrm{ EF}i$$

where, E_i_ is mass (in g) of emitted species *i*; A is area burned (in m^2^) at grid cell *x*; FL is total fuel load (in kg m^−2^) at grid cell *x*; CC is combustion completeness (or the fraction of consumed fuel, 0–1 scale); and EF*i* is the emission factor (in g kg ^−1^) of the chemical species *i*.

Our study focused on the spatiotemporal variability of CO_2_ and PM_2.5_ emissions given their crucial role in regulating the earth’s carbon and energy budget, and the latter influencing human health; as such, other CO_2_-equivalent gases (methane and nitrous oxide) were not considered. Likewise, the choice of PM_2.5_ over PM_10_ was guided by the fact that for biomass combustion emissions, PM_2.5_ makes up the majority of PM_10_, and is more damaging to human health than PM_10_. The pollutant can penetrate the lungs and be transported to other organs through the bloodstream and trigger reactions such as bronchitis, asthma attacks, cardiovascular diseases and premature mortality [36].

Area burnt records, obtained from Tasmania Fire Service, included unburnt patches within the fire perimeter. These were excluded from the analysis so that only burnt areas remained, covering 20,200 ha of the perimeter. Fuel load estimates representative of all vegetation within the perimeter were absent except for one site that was sampled after the fire from paired burnt-unburnt plots [37]. We therefore adopted fuel load estimates (in t ha^−1^ dry matter) across Tasmania and from literature on southern *Eucalyptus* forests of Australia (Table 1). Since a large variability of fuel loads existed across different regions in Australia, emissions calculation involved a bootstrapping of all available ranges of fuel load within each vegetation class to account for the uncertainties propagated by fuel loads. Fuel loads in Table 1 have been stratified into fine (diameter < 0.6 cm) and coarse woody debris (CWD, diameter > 0.6 cm), where fine fuels represent surface to elevated fuels (e.g., litter, standing herbs, grass and fine twigs), while CWD represents fallen twigs, branch wood, logs and stumps.

**Table 1:** The variability of fuel load (in t ha^−1^ of dry matter) within the general southern Australia *Eucalyptus* forests

Parameter		Min.	Mean	Max.	Notes and references
Fuel load (t ha^−1^)					
Dry forest	Fine	10	21	34	From unpublished records from Tasmania Fire Service (TFS)
		9	14	21	For Tasmania where minimum value represents fuel age > 10 years. Maximum is for the maximum possible estimates in SE Tasmania [74]
		–	9	–	Recommended for Tasmanian woodlands [45]
		–	–	25	Maximum potential values for dry forest (shrubby/grassy) of New South Wales [75]
	CWD	22	74	175	From Hollis et al. [76]
		5.1	50	221	From Woldendorp and Keenan [77]
		–	16	–	Recommended for Tasmanian woodlands [45]
Wet forest	Fine	10	31	41	From unpublished records from Tasmania Fire Service
		–	9	–	Recommended for Tasmanian forests [45]
		–	11	–	From un-thinned sites in *E. delegatensis* forests
		1	10	–	From unburnt sites in Victorian obligate seeder forests [78]
		–	–	39	Maximum potential values for wet forest (shrubby) of New South Wales [75]
	CWD	49	86	123	From Hollis et al. [76]
		0.2	134	1089	From Woldendorp and Keenan [77]
		–	14	–	Recommended for Tasmanian forests [45]
			23	–	From un-thinned sites in *E. delegatensis* forests [79]
		–	11	–	From unburnt sites in Victorian obligate seeder forests [78]
Hardwood plantation	Fine	17	19	–	From unpublished records from TFS
		3	14	39	Measurements from Australian forests [77]
	CWD	1.2	10	49	From Woldendorp and Keenan [77]
Softwood plantation	Fine	16	18	–	From unpublished records from TFS
		–	–	26	Represents 80th percentile of total fine fuel in exotic plantations in Queensland, Australia [80]
	CWD	3.1	67	144	From Woldendorp and Keenan [77]
Non-forest	Fine	4	8	24	From unpublished records of grasslands from TFS
		0.88	2^a^	12	For Tasmanian native grasslands [81]
	CWD	–	1	–	Estimates assumed to be half the amount of fine fuels from native grassland

*Fine* fine fuel, *CWD* coarse woody fuel. ^a^Represents the median fuel load estimate.

Since CWD fuel loads identified in the literature included outlying extreme values in the native forests, some of which were obtained following logging operations and included exaggerated coarse debris, we decided to use variability of mean values of fine and coarse fuels within each vegetation type to limit the influence of these outlying values. We conducted 100 simulations where within each run, all grid cells for a given vegetation type were assigned the same random fuel load value drawn from a uniform distribution from the available range of mean fuel load values (Table 1). For example, in any one simulation, all cells within the dry forest class were assigned a similar fine fuel value between 9 and 21 t ha^−1^, and CWD value between 16 and 74 t ha^−1^, with the values changing for every simulation so that at the end, 100 emissions estimates are produced. The fuel load values were converted to kg m^−2^ and aggregated to 2500 m^2^ to harmonise all analyses at a 50 m × 50 m grid cell scale.

Combustion completeness (fraction of fuel burnt) was determined based on a combination of previous fire severity mapping for this study area [33] and field measurements of fuel consumption in prescribed and wildfires in *Eucalyptus*-dominated forests in Tasmania and south-eastern Australia (Table 2). We chose these data sources to estimate fuel consumption because field measurements of consumption after the Forcett–Dunalley fire were largely lacking. We partitioned fuel consumption according to severity classes mapped from the Forcett–Dunalley fire based on the assumption that areas with high fire severity have most (or all) of the fine, coarse dead fuels and canopy burnt while for areas that burnt under mild severity, a lower fraction of the fuel mass is consumed (Table 2). The CWD combustion estimates in Table 2 concur with woody fuel consumption estimates reported by Hollis et al. [38] in two high-severity fires: the Kilmore East fire and the Pickering Brook fire. Fire patchiness, which is usually incorporated in the estimation of combustion efficiency, was assumed in this study to be accounted for by the high spatial resolution of the severity mapping. Therefore, patchiness at a resolution below that of the pixel dimensions was not considered. No differentiation in fuel consumption is made between different woody vegetation classes (native or plantation forest). We acknowledge the lack of site-specific fuel consumption also introduces uncertainties in estimation of emissions [39, 40].

**Table 2:** Estimates of consumed biomass per fuel size class and fire severity (dNBR) class for native (dry and wet *Eucalyptus* forests) and plantation (*Pinus* and *Eucalyptus*) forests, obtained from previous field measurements of native forests in Tasmania and mainland Australia

Vegetation class	Severity	Consumed fuel (0–1)		References and notes
		Fine	Coarse	
Native and plantation forests	Low	0.6	0.25	From Volkova and Weston [20] for prescribed burns; as well as from one paired burnt-unburnt field plot for this study area [37]
	Medium	0.8	0.46	From Hollis et al. [41] as average consumption across plots from regeneration burning in Warra, Southern Tasmania
	High	1	0.65	From O’Loughlin et al. [42] for severe fires under moderate drought. CWD estimate is the mean of 5–10 cm diameter branches (74% consumed) and 20 cm logs (56% consumed)
	Very high	1	0.9	CWD estimate based on consumption in high fire severity (CBI of 2.45) plots in Tasmania, and from a severe crown fire in Volkova et al. [43] and [38]
Non-forest	^a^Very high	1	0.72^b^	Recommended by Environment Australia [44] for wildfires in temperate grasslands

^a^Fire severity for non-forest class from aerial photography interpretation of the Forcett-Dunalley fire was very high as the fire burns all the aboveground biomass, although biological impact is obviously not comparable to woody vegetation

^b^The recommended value (0.72) is assumed to represent coarse fuels. CBI is Composite Burn Index, a field-based assessment of fire severity commonly used in coniferous-dominated vegetation in North America

Lastly, we adopted emission factors for CO_2_ and PM_2.5_ from literature based on lab analysis and previous prescribed burning campaigns in southern Australian *Eucalyptus* forests (Table 3). Emissions factors have not been partitioned into different vegetation classes because estimates are lacking in most classes found in the study area.

**Table 3:** Emission factors (in g kg^−1^) for CO_2_ and PM_2.5_ for fine and coarse fuels as used in Southern Australian *Eucalyptus*-dominated landscapes

Emitted pollutant	Emission factors		References
	Fine	Coarse	
CO_2_	1730	1514	Roxburgh et al. [45]
PM_2.5_	16.9	38.8	Reisen et al. [26]

EF for CO_2_ represents the mean EF harmonised in Roxburgh et al. [45] from previous studies of EFs in *Eucalyptus* forests of Australia

### Spatiotemporal distribution of emissions

**Fig. 2:**
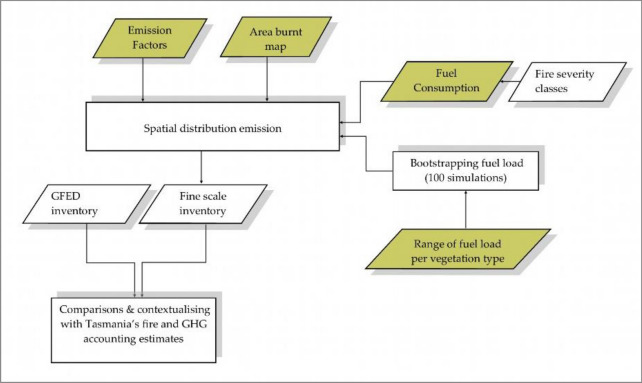
Systematic flowchart of emissions analysis from the Forcett–Dunalley fire, with inputs obtained from available geospatial datasets, previous field assessments and literature

The spatial distribution of emissions was determined by combining the aforementioned model variables in Eq. 1 [34] using R version 3.6.1 [46] and ArcGIS 10.3 [47]. The fine scale approach (using 50 m grid resolution) followed a schematic workflow (Fig. 2), which includes the mentioned input variables in Eq. 1. A feature of this analysis is the use of detailed fire severity information and vegetation mapping to estimate emissions. Maps of the spatial distribution of emissions of both CO_2_ and PM_2.5_ were produced, where estimates in each grid cell were totals from emissions values for both fine and coarse fuels. Total emission for each pollutant was determined for each of the 100 runs by summing values from all grid cells. We then obtained a bootstrapped mean and standard deviation of total emissions across the runs.

A daily variation of these emissions was determined by intersecting the final emissions map with the fire progression isochrones and summing emissions contained within each temporal polygon. It is worth noting that at the start of the fire, the fire spread polygons were available at sub-daily intervals but as the fire progressed, the time interval between available boundary mapping increased to day(s). We therefore aggregated emissions from sub-daily resolution to daily progressions by combining all emissions for each day.

#### Comparison with GFED inventory

To assess the reliability of a global emissions model (GFED) in situations of unavailability of site-specific fire data, we compared the spatial and temporal variability of CO_2_ and PM_2.5_ emissions between the above fine scale analysis and the GFED4 inventory for January 2013. GFED4 is an industry-standard global emissions model that provides 3-hourly, daily and monthly estimates of 42 emissions species from across the globe at 0.25° (~ 28 km) spatial resolution from the year 1997 [48]. GFED is based on a Carnegie–Ames–Stanford Approach (CASA) biogeochemical model that simulates carbon fluxes from satellite-based observations of vegetation, weather, area burnt and combustion completeness. A full description of the model is provided in van der Werf et al. [48].

We downloaded two gridded datasets (combusted dry matter (DM), and the area burnt layer for January 2013) from the GFED website [49], and multiplied the variables with recommended GFED emission factors for temperate forests (12.9 and 1647 g kg ^−1^ for PM_2.5_ and CO_2_ respectively). The result was a spatial map of the two emissions for the entire Tasmania, and a monthly estimate for January 2013 for specific cells that represent the Forcett–Dunalley fireground. These monthly estimates were partitioned into daily emissions by using a daily fraction file that contains the contribution of each grid cell to the total emissions. The daily and spatial variation of the resulting maps from the fine scale and GFED inventories were quantitatively and visually compared to determine the effect of the geographic resolution of fire severity and vegetation mapping on emissions. It should be acknowledged that the daily GFED estimates were only available for 3–14 January, which coincide with the duration of MODIS thermal hotspots data available for the study area. It is therefore likely that the burnt area layer was obtained from a combination of spectral reflectance of burnt area and thermal hotspot data, the latter of which is adopted in GFED4 to represent small fires that would have been missed in previous GFED versions.

We then validated the two emissions inventories using FullCAM simulation of carbon emission (which can be converted to CO_2_ via 3.67 factor) over the Forcett–Dunalley fireground. FullCAM is a modelling interface used in Australian GHG accounting of the land sector [50], and can simulate fire emissions as an event by feeding in carbon flux estimates from combustion of forest debris and live biomass. Major emission outputs of the model include methane, nitrous oxide and carbon. To determine carbon emissions within FullCAM, we used input parameters values recommended in Surawski et al. [50] for wildfire events with fire intensities of > 7000 kW m^−1^ in which trees have not been killed.

### Contextualising emissions in Tasmania

To gauge the relative contributions of the Forcett–Dunalley fire (that included a significant PyroCb event) to typical annual fire emissions in the state, we compared the Dunalley emissions with the mean fire emission estimates for Tasmania for the period 1997–2020 (the period of the available GFED record). First, we merged GFED estimates across the different vegetation types in Tasmania to produce an annual emission estimate for the above period. Since the GFED emissions were available as carbon emissions, for comparison with estimates from Dunalley fire, we converted GFED’s carbon emissions estimates to CO_2_ (using 3.67 conversion factor). The percentage of Forcett–Dunalley emissions was then estimated relative to: (1) the total 2013 fire emissions across the state, and (2) mean annual fire emissions for the state. We then examined Tasmania’s fire emissions relative to the state wide carbon (GHG) emissions budget, in order to quantify the effect of excluding severe fires from GHG accounting under the assumption that the fires are natural disturbances and beyond human control.

## Results

### Spatial distribution of emissions

**Fig. 3:**
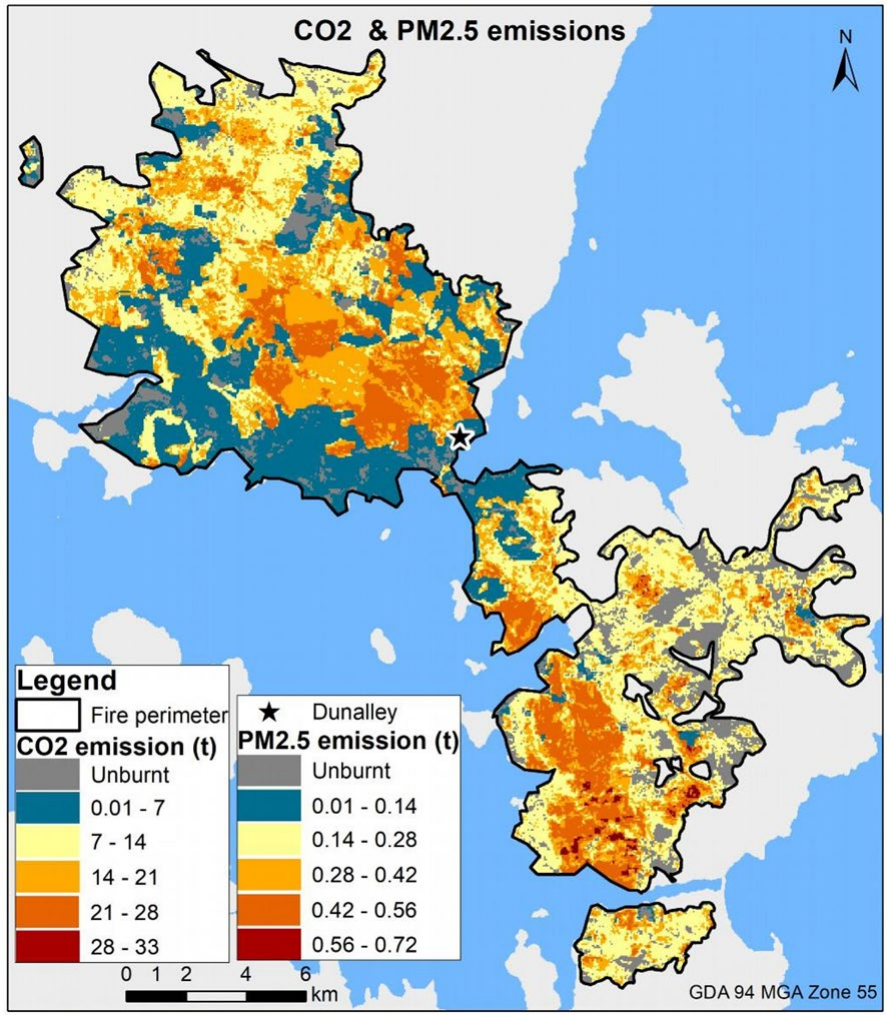
Spatial distribution of CO_2_ and PM_2.5_ emissions (in tonnes per 50 m grid cell) from the Forcett–Dunalley fire as a bootstrapped mean of total emissions per grid cell, from the 100 simulations. Note the similarity in emissions patterns for the two emissions

From the fine scale emissions inventory, total CO_2_ emissions were 1.125 ± 0.232 million tonnes (or 1.125 ± 0.232 Tg), translating to 55.7 t ha^−1^ of CO_2_ released from the 20,200-ha burnt area (Table 4). PM_2.5_ emissions reached 0.022 ± 0.006 Tg and 1.1 t ha^−1^ when normalized by area burnt. Carbon dioxide emissions varied across the fireground, reaching 33 tonnes per 50 m resolution grid cell, while the PM_2.5_ emission peaked at 0.72 tonnes (Fig. 3). It is worth noting that the spatial patterns of both CO_2_ and PM_2.5_ are identical because they are based on the same amount of consumed fuel per unit area, but only differ in their respective emissions factors. In both pollutants, the highest emissions were in the south-southwest of the fireground, characterized by the highest fire severity classes (Fig. 1d). These areas also coincided with a large flaming zone in the classified infra-red linescan map for 4 January (see Additional file 1: Fig. S1) which was associated with elevated fire weather.

**Table 4:** Total CO_2_ and PM_2.5_ emission, and emissions standardized by burnt area from the Forcett–Dunalley fire

Model	CO_2_ emission		PM_2.5_ emission	
	Total (Tg)	Standardized (in t ha^−1^)	Total (Tg)	Standardized (in t ha^−1^)
Fine scale	1.125 ± 0.232	55.7	0.022 ± 0.006	1.1
GFED4	0.822	36	0.006	0.3

The standard deviation around the bootstrapped mean of total estimates are provided for the fine scale inventory

**Fig. 4:**
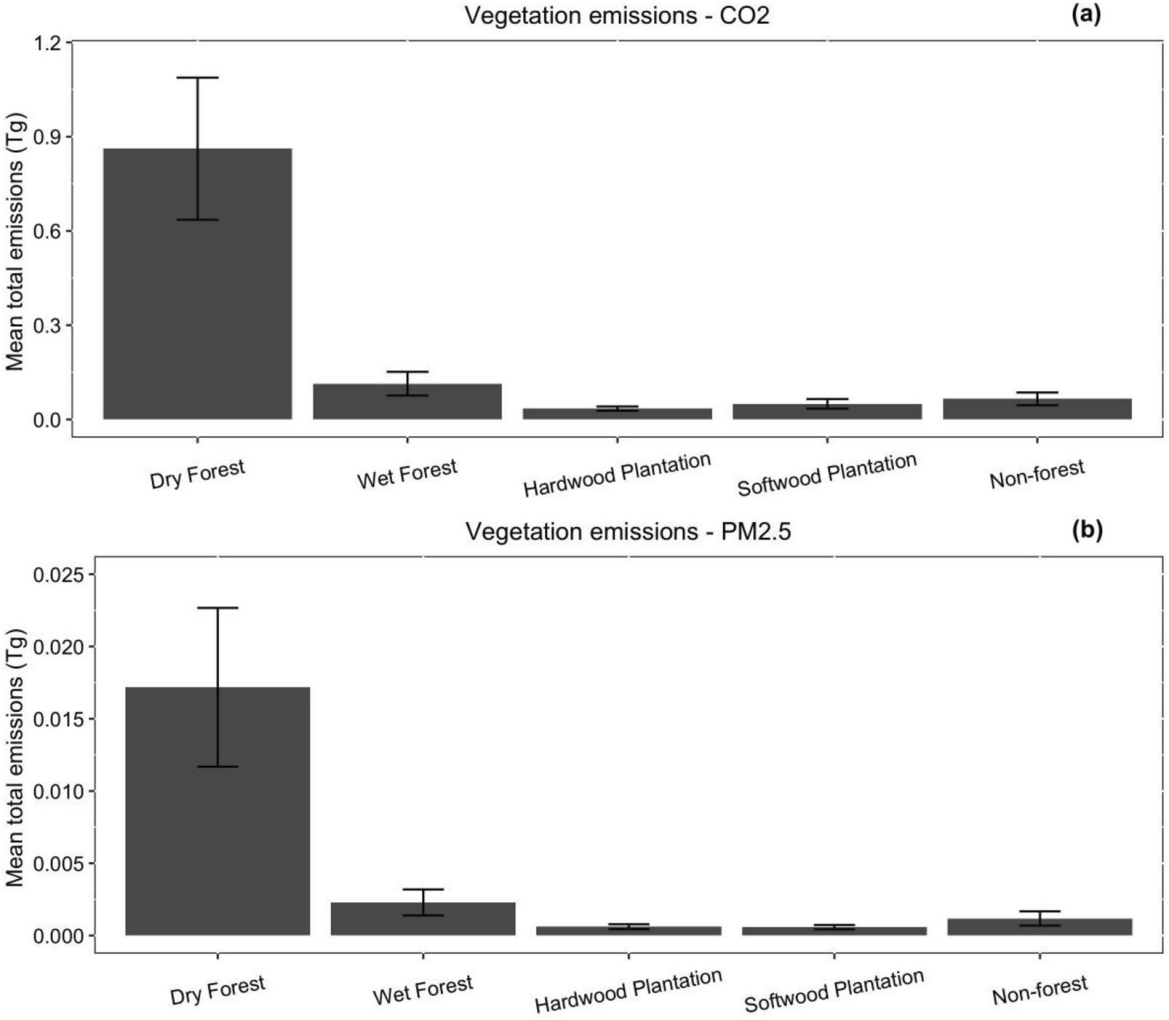
Bootstrapped mean and variability of total emissions from the different vegetation types found within the fireground. **a** represents CO_2_ emissions and **b** PM_2.5_ emissions

Overall, the dry forest contributed the highest proportion (77–79%) of total CO_2_ and PM_2.5_ emissions respectively, while the wet forests contributed approximately 10% of both emissions (Fig. 4). This reflects the greater proportion of dry forests in the area burned at higher intensity, although the highest variance was in the wet forest and *Pinus* (softwood) plantation (coefficient of variation of ~ 31% CO_2_ and ~ 40% PM_2.5_ for both vegetation classes), with only a few areas burning intensely. The emissions variability for the dry forest was around 26–32% for the two pollutants respectively, while the *Eucalyptus* (hardwood) plantation displayed the lowest variability, at 20–28% for the two pollutants respectively (Fig. 4).

### Model comparison

**Fig. 5:**
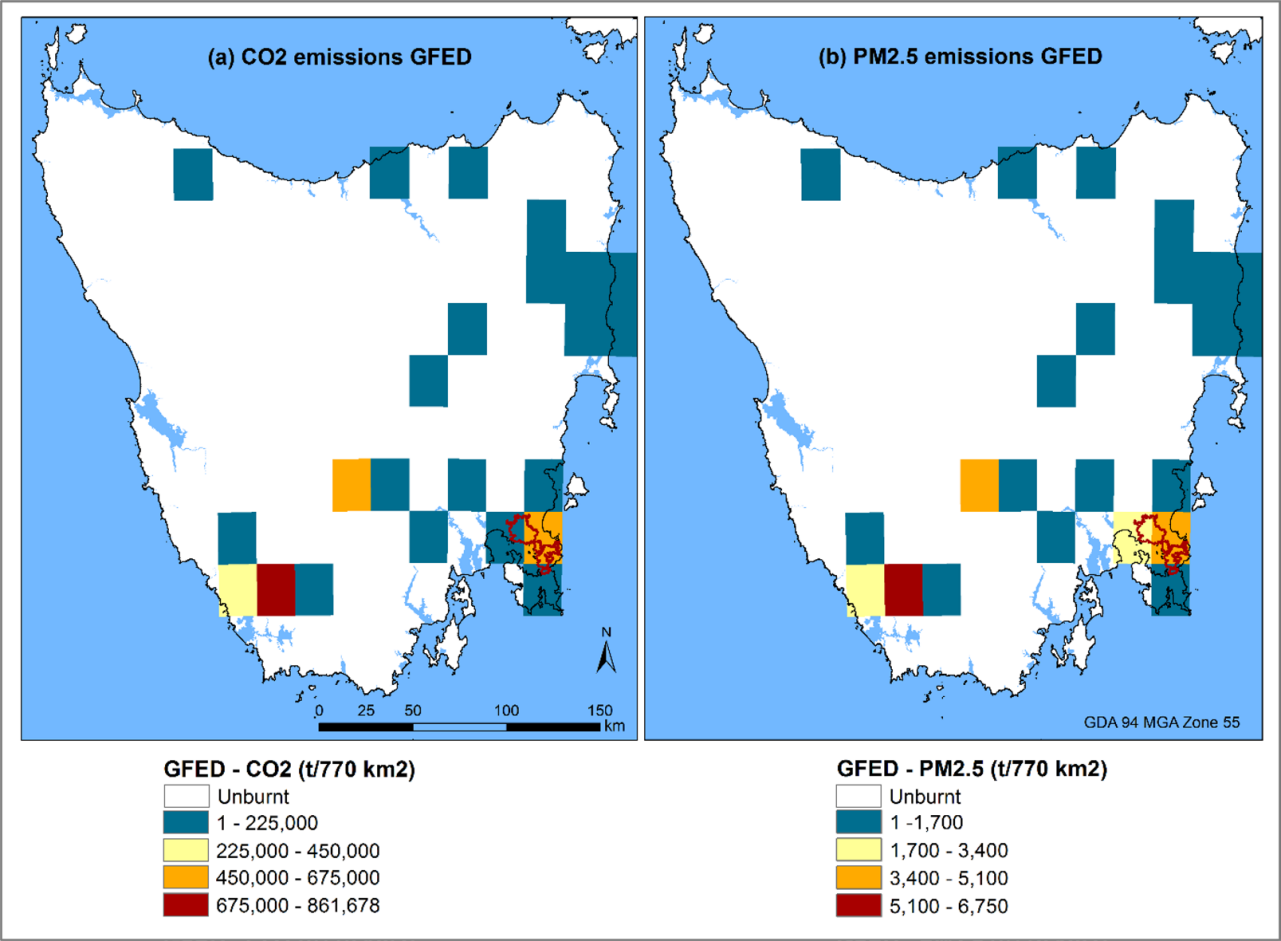
Spatial distribution of CO_2_ and PM_2.5_ emissions (in tonnes per 28-km grid cell) from several fires in mainland Tasmania, including the Forcett–Dunalley fire (red polygon) for the entire January 2013 from GFED4 analysis

The fine scale estimation, that incorporated detailed fire severity and vegetation mapping, had a better characterisation of the spatial variability of both emission types than GFED (compare Figs. 3 and 5). Nonetheless, GFED detected the area with the highest emissions, with an added advantage of providing a synoptic view of several fires burning across Tasmania. A comparison of total CO_2_ and PM_2.5_ emissions between the two inventories revealed comparable emissions estimates, especially for CO_2_ (Table 4). The fine scale analysis produced total CO_2_ emissions (and range) of 1.125 Tg (0.893–1.357 Tg) compared to GFED’s estimate of 0.822 Tg which is 73% (range of 65–92%) of the CO_2_ emissions estimate from the fine scale inventory. However, for PM_2.5_, GFED reported much lower emissions of 0.006 Tg relative to 0.022 ± 0.006 Tg from the fine scale analysis, representing 30% (24–41%) of the emissions estimate in the fine scale inventory. Per-hectare emissions were comparable but lower for GFED, with 36 t ha^−1^ for CO_2_ and 0.3 t ha^−1^ for PM_2.5_ (Table 4). It’s worth noting that the area burnt estimate from GFED was approximately 22,851 ha, which is similar to the area estimated by the fine scale analysis (20,200 ha).

**Fig. 6:**
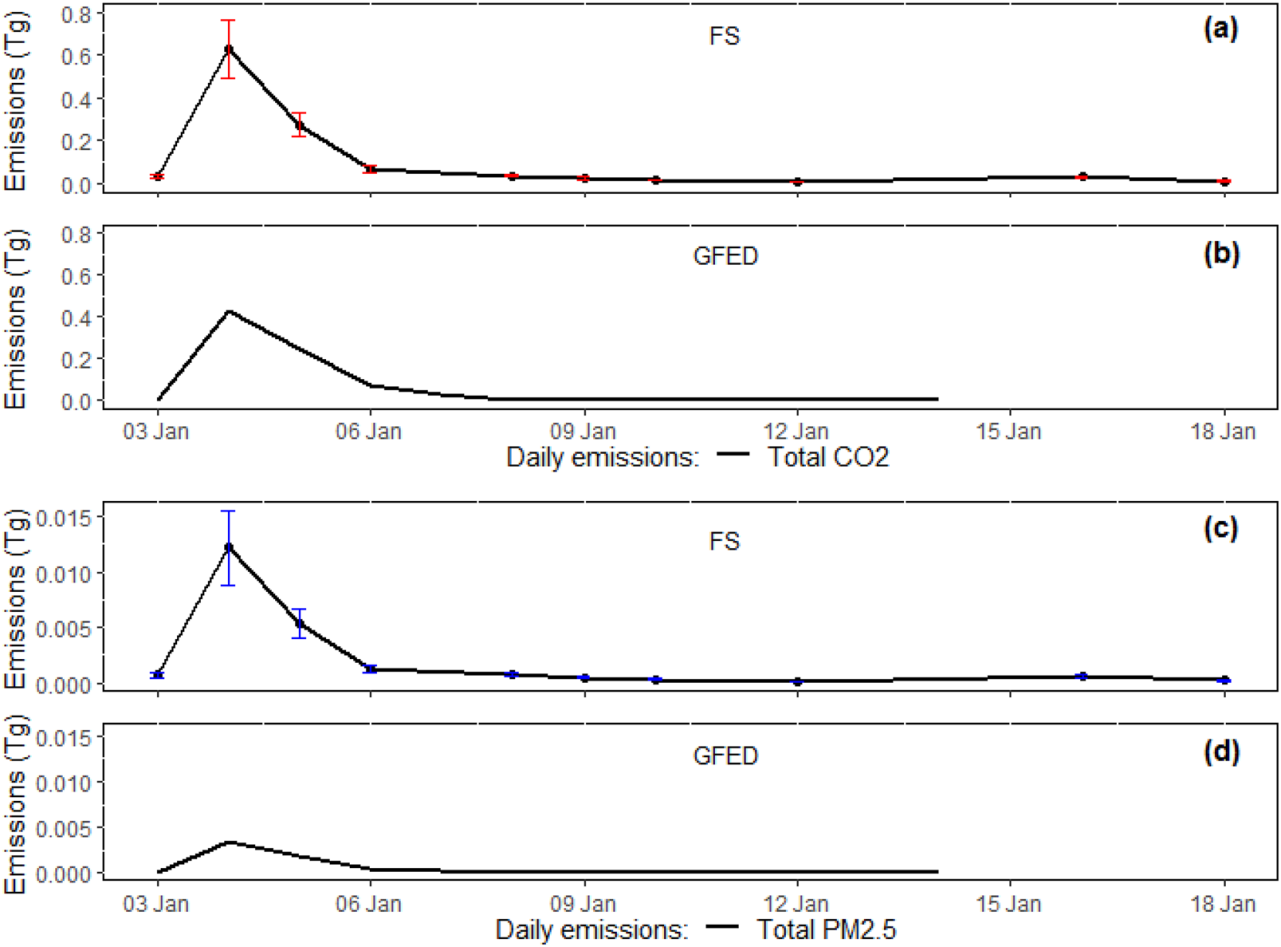
Daily variability of CO_2_ and PM_2.5_ emissions from the Forcett–Dunalley fire between the fine scale (FS) and GFED inventories. **a**, **b** Represent CO_2_ variability while **c**, **d** show PM_2.5_ variability for each of the inventories. The error bars represent the standard deviation values around the mean of bootstrapped total daily emissions. 4 January is the day of the PyroCb occurrence

The GFED estimates for the study area were only available until 14 January 2013 and during this period, temporal variability of the two emissions showed similar trends between the fine scale and GFED inventories (Fig. 6). These trends were significantly correlated (*r* = 0.99, *p* < 0.05), albeit emission estimates from GFED were always lower than the fine scale analysis. The 4 January had the highest emissions of all days, a day notable for the formation a pyrocumulonimbus (PyroCb). Emissions then drastically declined on 5–6 January and subsequently stabilised at lower values till containment of the fire.

Overall, burnt area mapping from GFED closely aligned with area estimates from the fine scale inventory; total emissions for CO_2_ were comparable in both inventories; with the models capable of capturing the temporal evolution of CO_2_ and PM_2.5_ emissions. However, validation of both inventories using FullCAM simulation over the Forcett–Dunalley fireground yielded approximately 38.6 t ha^−1^ of carbon emission (or 142 t ha^−1^ of CO_2_), which is more than twice the estimates from both inventories.

### Fire emissions in Tasmania

**Fig. 7:**
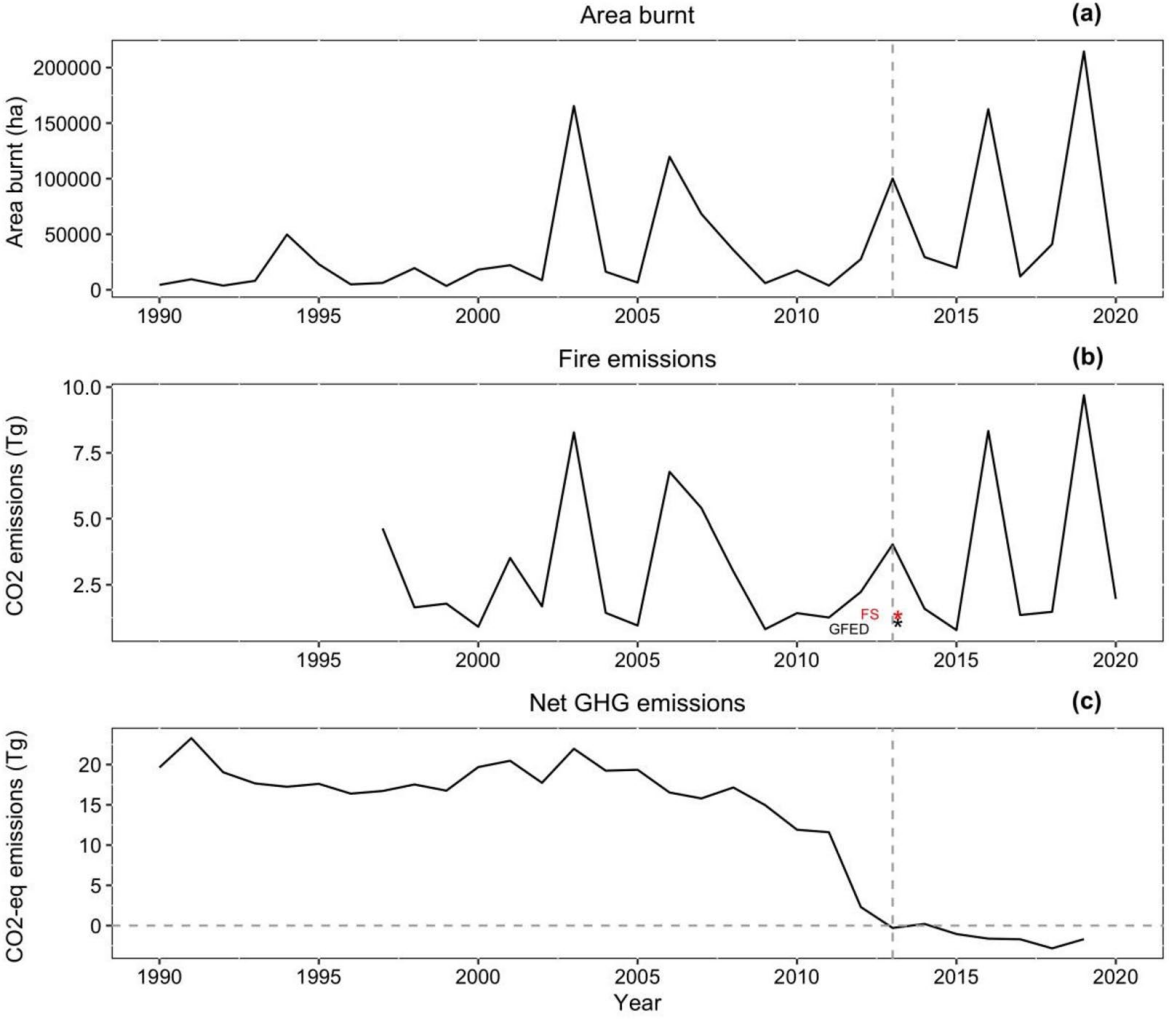
Time series of carbon emissions across Tasmania for the period 1990–2019. **a** interannual variability of area burnt within the state; **b** variability of total annual wildfire emissions based on the available GFED record; and **c** interannual variability of GHG (CO_2_-equivalent) emissions according to the State’s Greenhouse Gas Inventory for 2019 that includes the period 1990–2019

Wildfire-derived carbon dioxide emissions and area burnt across Tasmanian fires revealed an interannual variability (Fig. 7a and b), both showing a similar trend where more emissions were produced with an increased area of unplanned fire (correlation of 0.925). Further, correlation for all fires combined (both planned and unplanned) was 0.891 although emissions from planned fires were negatively correlated with area burnt (*r* = − 0.203), suggesting that increased planned fire area slightly reduces CO_2_ emissions. Conversely, the wildfire emissions trends do not correlate with Tasmania’s GHG (CO_2_-equivalent) accounts (Fig. 7c), which show a sharp decline in GHG emissions in 2012 and a stable reduction in the after-years (to being net carbon sink from 2013) despite a spate of large Tasmanian fires in 2013, 2016 and 2019. It is worth noting that fire emissions for the period January-March 2019 are missing from the GFED record, a period characterised by extensive wildfires. It is likely that fire emissions for year 2019 are considerably underestimated.

From the wildfire-related estimates in Fig. 7b, the Forcett–Dunalley fire represented 28% (almost a third) of fire emissions in Tasmania during the 2013 fires, and 36% and 26% of mean annual fire emissions (3.12 Tg CO_2_) for the period 1997–2020, based on fine scale and GFED estimates respectively.

## Discussion

This study adopted a ‘bottom-up’ emissions methodology to quantify CO_2_ and PM_2.5_ emissions from the 3–18 January 2013 Forcett–Dunalley fire in south-eastern Tasmania. We show that total CO_2_ and PM_2.5_ emissions from the fine scale analysis reached 1.125 ± 0.232 Tg and 0.022 ± 0.006 Tg respectively. A comparison of the fine scale (50 m) analysis that uses local fuel and fire severity estimates, and a coarse scale global emissions model GFED (0.25 degrees or ~ 28 km) showed that GFED had a good agreement with the fine-scale analysis regarding total CO_2_ emissions but not PM_2.5_ emissions. Naturally, fine scale analysis had more detailed spatial patterns of both emissions than GFED. Validation of the emissions estimates using the FullCAM model yielded 142 t CO_2_ ha^−1^ (> 2 times the estimates from both inventories), suggesting that further refinement of FullCAM is important, especially the parameters used in calibrating the model (e.g., debris pool) which are subject to large uncertainties [50].

### Other wildfire emissions

A comparison of Forcett–Dunalley fire emissions with other Australian temperate fires showed similarities with some fires and considerable differences with other fires (see Additional file 2: Table S1). For example, the per-hectare CO_2_ estimate from this study was 55.7 t CO_2_ ha^−1^ whereas Volkova et al. [43] reported emission of 105 t CO_2_ ha^−1^ from a wildfire in a long-unburnt dry shrubby *Eucalyptus* forest in Victoria. However, our values are comparable to those reported by these authors from the areas within that wildfire that were previously fuel-reduced (42 t ha^−1^ of CO_2_). The 2003 Canberra fire produced 20.2 Tg of CO_2_ emissions based on the Australian FullCAM model [51], translating to approximately 78 t CO_2_ ha^−1^ from the 260,000 ha-fire size, assuming no unburnt patches. However, other studies have reported carbon emissions estimates of 40 M tonnes (or 40 Tg) from the same fire [52]; it is likely that CO_2_ emissions from that fire exceeded 400 t ha^−1^ given that CO_2_ emission are 3.67 times more than carbon emission.

Previous studies in Australia have shown high agreement between GFED and other models/field observations in CO_2_ emissions e.g., Paton-Walsh et al. [24]. This is despite GFED treating vegetation types, particularly *Eucalyptus* forests and woodlands, and fire behaviour in south-eastern Australia as the same as those found in the temperate biomes in Northern Hemisphere. The overall good performance of GFED’s CO_2_ estimates in this study also likely reflects an improved detection of smaller fires in GFED4 compared to previous versions of GFED [48].

Per-hectare estimates for PM_2.5_ in this study (1.1 t ha^−1^) were inconsistent with emissions estimates from other Australian temperate fires (Additional file 2: Table S1). For example, Reisen et al. [26] reported emissions of 73.7–163.9 kg ha^−1^ (0.07–0.16 t ha^−1^) from prescribed fires in Victorian *Eucalyptus* forests while another Tasmanian study reported PM_2.5_ emissions of 7789 tonnes (or 6.9 t ha^−1^) from a high-intensity regeneration fire in a southern Tasmanian native forest [53]. It should be noted that there is paucity of data on PM_2.5_ emission from temperate Australian forest fires; most of the studies have instead focused on PM_2.5_ concentration in urban airsheds for air quality purposes, involving a mix of emission sources. Beyond Australia, western US wildfires between 2011 and 2015 were estimated to have emitted 1530 Gg (1.53 Tg) of PM_2.5_ annually [54]. Similar to our study, the authors report that the emissions were three times higher than the estimates from the US national inventory. Further, in another study, the GFED3 PM2_.5_ emission estimate across contiguous US was lower by a factor of eight compared to the national emissions inventory [55], revealing a likely systematic underestimation of PM emission across jurisdictions.

### Deficiencies in current fire emissions approaches

The discrepancy in GFED modelling in this study was the lower PM_2.5_ emissions by a factor of three, likely due to lower emissions factors (EFs) used for PM_2.5_ within GFED (12.9 g kg^−1^). These EFs do not accurately reflect temperate *Eucalyptus*-dominated fuels in Australia, as they are averaged across the temperate biome globally. One of the main differences significantly affecting emissions amongst the temperate biomes is fire behaviour. For example, compared to other biomes, Australian forests and woodlands typically have a higher biomass of sclerophyllous leaves and bark, which burn intensely and support short-long distance transport and spotting of embers that spread landscape fire [56]. *Eucalyptus* fuels have lower rates of decomposition (and therefore low/absent duff layer [57]) compared to northern hemisphere conifer/boreal forests that have a more-developed duff layer that supports smouldering combustion and can contribute up to 50–74% of fuel consumption [58]. An upward revision of PM_2.5_ EFs to 16.9–38.8 g kg^−1^ [26] is therefore recommended to better accommodate typical fuels within these Australian ecosystems.

The accuracy of bottom-up approaches (such as the above inventories) that adopt fuel consumption estimates in emissions estimations has been a topic of debate relative to the more accurate top-down approaches that use satellite observations to directly estimate emissions within the atmospheric column [59–61]. Despite these limitations, two previous carbon emissions studies on the recent Australian Black Summer fires using top-down and bottom-up approaches revealed comparable CO_2_ estimates between the two methods [3, 13]. This highlights the importance of validating emissions estimations with diverse methods, including satellite and on-ground observations, to reduce the inherent uncertainties.

Smoke emissions analyses are constrained by the quality and representativeness of data on fuel types, requiring greater sampling of a broader range of vegetation and fuels [20]. Field protocols should include detailed inventories of vegetation characteristics, e.g., Prior et al. [62] and measurement of fuel loads across all fuel components, ranging from subsurface to overstorey fuels, and from fine to woody fuels. To date, coarse woody debris (CWD) estimation, being the less studied fuel component than fine fuels, is the most common source of emissions uncertainties in temperate Australian landscapes. This is because CWD is influenced in different regions by among other factors, the disturbance history (past fire or logging activities), forest age, and site productivity [18, 63]. More field inventories across Australia and particularly in Tasmania where there has been scarcity of fuel load data [45] are needed to provide confidence in emissions estimates.

Fire behaviour modelling in Australia has shifted from an emphasis on fine fuel loads, to a more realistic determination of fuel hazard scores across fuel types; nonetheless, we contend that there remains a need for accurate fine and coarse fuel load measurements to underpin fire emissions analysis [64]. These inventories could make use of recent technologies such as LiDAR to increase the accuracy of fuel estimation, especially the amount of coarse woody debris, within a forest. Previous research has shown that carbon losses from forest regeneration burns are around 200 t ha^−1^ [65]. However, the relationship between forest harvesting and likelihood of uncontrolled fires, that would cause higher carbon emissions than if native forests were unharvested, is highly controversial and demands further research [66, 67]. Another important knowledge gap concerns the comparative assessment of particulate and carbon emissions and associated costs of fuel management burns, post-logging (or regeneration) burns and wildfires. Previous research into health economics suggests the public health cost of both fuel management burns and wildfires can be substantial [68].

Fire severity scales with fuel consumption, with high-severity fires typically associated with high consumption of vegetation; however, the general lack of empirical fuel consumption data can introduce variability in total emissions, despite the availability of fire severity information. This was evident in the spectral signatures (from satellite observations) in grassland areas of the Forcett–Dunalley fireground which exhibited very high severities despite their very low fuel loads and minimal biological impact. Fuel consumption estimates in this study were inferred from a few studies on temperate *Eucalyptus* forests (Table 2). Therefore, there is need to improve data collection of fuel consumption during wildland fires (supplemented by remote sensing), and measurement of residence time of flaming and smouldering to partition emissions into the different combustion stages. Although these attributes can be inferred from lab experiments, variability in fuel size, especially coarser fuels are difficult to accurately characterise in the lab [69]. There is also a need to clearly establish a quantitative link between severity measurements and fuel consumption for better applicability of fire severity data in future emissions studies.

### Greenhouse gas accounting

Estimates of emissions from wildfires are of increasing interest given their contribution to climate change. Indeed, emissions from Australian wildfires are accounted for in the national GHG accounting to the Intergovernmental Panel on Climate Change, however, what constitutes a wildfire and a human-caused fire in the accounting is subject to debate and a number of pragmatic and often poorly justified ‘rules’. For example, the Australian Government accounting uses a burned area threshold (that is 16,950 ha in Tasmania) and fire emissions threshold (2 standard deviations above the mean of gross annual fire emissions) to exclude large fires or fire years, with the assumption that the fires were not human-caused and therefore are under no human control [29]. These statistically large fires are therefore attributed as natural disturbances and are excluded in the final carbon accounting, in the same way post-logging regeneration fires are excluded. It is therefore likely that the Forcett–Dunalley fire (with a burnt area of > 20,000 ha was excluded based on these criteria despite it being anthropogenically-caused. While there is some logic to this reasoning, there is uncertainty as to how to treat severe wildfires, such as the Dunalley disaster, that are human-caused, are exacerbated by anthropogenic climate change, burn over a highly human-modified landscape, and are subject to intensive human control efforts, yet they exceed the above threshold for defining anthropogenic fires.

Although, it is commendable that from the year 2019, the Australian government can report to IPCC on fire emissions within the ‘natural disturbance’ provision [51], we recommend inclusion of all emissions from large, human-caused fires as well as post-logging burns at state and national levels in the final accounting, to prevent situations where net carbon credits are claimed despite insufficient fire management. Current accounting approaches can potentially lead to perverse outcomes where carbon neutrality could be claimed by reducing the extent of planned fires that are an important tool in mitigating uncontrolled bushfire and reducing emissions (Fig. 7). Current arrangements therefore provide disincentives to effective wildfire management to reduce carbon emissions from large wildfires and post-logging fires that ultimately exacerbate climate change. Furthermore, the national policy is inconsistent because in north Australian savannas, there are carbon emissions abatement programs which reward pre-emptive early dry season burning to limit the high smoke emissions associated with late season burning [70].

Tasmanian government’s GHG reporting reveals that since 2012, forestry-related activities (LULUCF) have counteracted anthropogenic non-forestry GHG emissions [71, 72], with an average removal of − 9.17 Tg between the years 2012–2019, and an increased carbon sequestration from − 5.920 Tg in 2012 to − 10.04 Tg in 2019. These estimates seem impressive; however, they are unaffected by major wildfires such as Dunalley disaster that according to the GFED model, accounted for one third of the state’s annual fire emissions. If severe-fire emissions were incorporated in the forestry-related GHG accounting for 2013 (− 10.952 Tg in forest land), Dunalley CO_2_ emissions (1.125 Tg) could have reduced forest land CO_2_ sequestration (or removal) by 10%. These results suggest that if wildfire emissions are included, then Tasmania may not be actually achieving carbon neutrality.

An important consideration in the understanding and accounting of carbon emissions is the influence of climate change on, and feedbacks with, fire regimes. In the GHG accounting across many national jurisdictions, the emitted carbon from wildfires is assumed to be assimilated by forests in the following growing seasons via tree growth, and therefore carbon uptake post-fire can be substantial. However, it is not clear how the regrowth and carbon sequestration can be relied upon in a changing hotter or drier climate. For instance, a warming earth has increased the vulnerability of ecosystems to frequent and intense fires, which in turn emit large quantities of emissions, thereby creating a positive feedback loop where forests are converted to a treeless state [73]. This calls for more investigation using diverse tools ranging from experiments, observations and models, to understand the complex interactions between climate, ecosystem structure and fire dynamics.

## Conclusion

This study quantified CO_2_ and PM_2.5_ emissions from the January 2013 Forcett–Dunalley fire using two standard emissions inventories. We report the release of approximately 1.125 ± 0.232 Tg of CO_2_ and 0.022 ± 0.006 Tg of PM_2.5_ into the atmosphere using a basic model that incorporated local fuel attributes. We investigated the reliability of a global model GFED4 in emissions estimation assuming the absence of field data. Our findings show that both the fine scale and GFED inventories produced comparable estimates for CO_2_, although PM_2.5_ estimates were lower by a factor of three for GFED. We therefore show that GFED was able to produce reliable emissions estimates within the limits of emissions uncertainties, although the model did not accurately capture the spatial distribution of the two emissions. By contextualising these estimates with wildfire emissions and overall GHG accounting in Tasmania, we show that the fire injected approximately 30% of fire emissions during the 2013 fire season, and represented 25–34% of mean annual fire emissions from the state. These findings showed the influence of the extreme fire event to overall carbon balance for the state, although the Forcett–Dunalley fire appears to have been excluded from the state and national carbon accounting due to the criteria that excludes natural disturbances fires. Such exclusions could have a major influence on a national or local jurisdiction’s claim of carbon neutrality. This analysis also investigated knowledge gaps in emissions quantification in Australian temperate *Eucalyptus* forests. We show that fuel attributes, especially the amount of coarse wood fuels within a forest stand, and the fraction of fuel consumed, contributed the most to uncertainties in emissions estimates. More accurate fine-scale analyses demand improved data on fuel types and their emission factors.

## Supplementary Information

**Additional file 1: Figure S1.** Spatiotemporal progression of combustion. Spatiotemporal progression of combustion during the early days of the fire, from classification of infrared linescan imagery obtained from a Victoria DELWP aircraft. The 4 January displayed dynamic fire behaviour of all the days during the fire. The original 20-cm resolution imagery has been resampled after classification to fit the 50-m resolution of the analysis.

**Additional file 2: Table S1.** Comparison of total emissions (in Tg) and per-hectare emissions (in t ha^−1^) among wildfires in Australia. Comparison of total emissions (in Tg) and per-hectare emissions (in t ha^−1^) among wildfires in Australia. Burnt area estimates (BA; in ha) for each fire event are indicated in brackets. CO_2_-equivalent (CO_2_-e) emissions are totals from CO_2_, methane and nitrous oxide emissions. The estimate for the Forcett–Dunalley fire (this study) has also been compared with estimates from the FullCAM model that is used in Australia for national GHG accounting.

## Data Availability

The datasets supporting the conclusions of this article are available in the following open-source databases: Burned area (fire history) records were obtained from Tasmania’s LISTmap (https://listdata.thelist.tas.gov.au/opendata/, last accessed: 4 March 2022). Two gridded datasets used in GFED4s emissions estimation (combusted dry matter and burned area) were downloaded from GFED website (https://daac.ornl.gov/cgi-bin/dsviewer.pl?ds_id=1293, last accessed: 4 March 2022). Time series data on Tasmania’s carbon accounting was extracted from the State and Territory Greenhouse Gas Inventory 2019, covering the period 1990–2019. (https://www.industry.gov.au/data-and-publications/national-greenhouse-accounts-2019/state-and-territory-greenhouse-gas-inventories-data-tables-and-methodology, last accessed: 4 March 2022).
