# Supplementary material for: Carbon dioxide and particulate emissions from the 2013 Tasmanian firestorm: implications for Australian carbon accounting
Source: Carbon Balance Manag. 2022 May 26;17:7. doi: 10.1186/s13021-022-00207-9 (PMC9134655; doi:10.1186/s13021-022-00207-9)
Supplement: Supplementary file 1 — Additional file 1: Figure S1. Spatiotemporal progression of combustion. Spatiotemporal progression of combustion during the early days of the fire, from classification of infrared linescan imagery obtained from a Victoria DELWP aircraft. The 4 January displayed dynamic fire behaviour of all the days during the fire. The original 20-cm resolution imagery has been resampled after classification to fit the 50-m resolution of the analysis. [file 13021_2022_207_MOESM1_ESM.pdf]

Carbon dioxide and particulate emissions from the 2013 Tasmanian Firestorm: Implications for Australian carbon accounting

Mercy N. Ndalila[1], Grant J. Williamson[1], and David M. J. S. Bowman[1]

Additional File 1

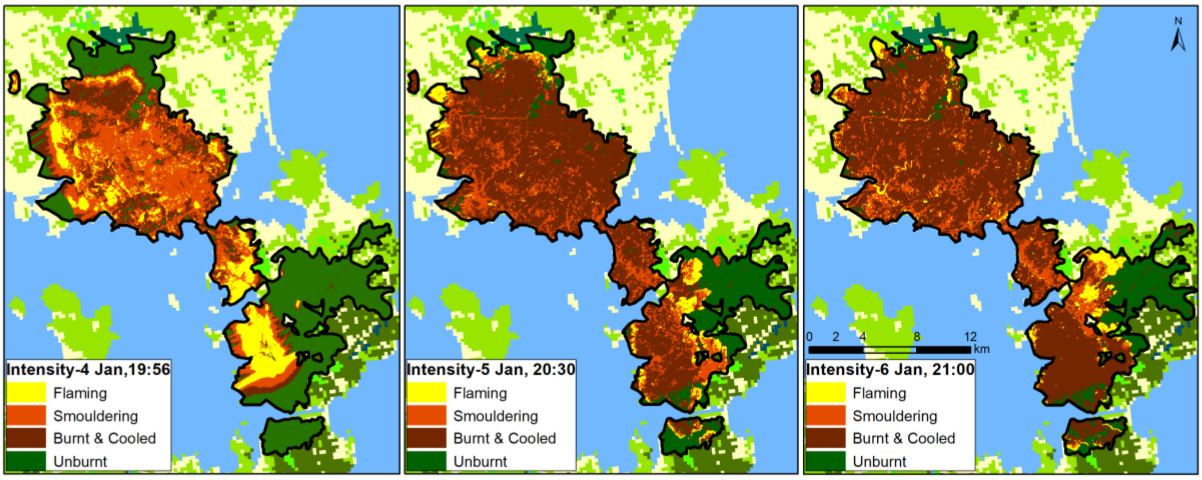

Figure S1: Spatiotemporal progression of combustion during the early days of the fire, from classification of infrared linescan imagery obtained from a Victoria DELWP aircraft. The 4 January displayed dynamic fire behaviour of all the days during the fire. The original 20-cm resolution imagery has been resampled after classification to fit the 50-m resolution of the analysis.
