# Supplementary material for: Carbon dioxide and particulate emissions from the 2013 Tasmanian firestorm: implications for Australian carbon accounting
Source: Carbon Balance Manag. 2022 May 26;17:7. doi: 10.1186/s13021-022-00207-9 (PMC9134655; doi:10.1186/s13021-022-00207-9)
Supplement: Supplementary file 2 — Additional file 2: Table S1. Comparison of total emissions (in Tg) and per-hectare emissions (in t ha−1) among wildfires in Australia. Comparison of total emissions (in Tg) and per-hectare emissions (in t ha−1) among wildfires in Australia. Burnt area estimates (BA; in ha) for each fire event are indicated in brackets. CO2-equivalent (CO2-e) emissions are totals from CO2, methane and nitrous oxide emissions. The estimate for the Forcett–Dunalley fire (this study) has also been compared with estimates from the FullCAM model that is used in Australia for national GHG accounting. [file 13021_2022_207_MOESM2_ESM.pdf]

Carbon dioxide and particulate emissions from the 2013 Tasmanian Firestorm: Implications for Australian carbon accounting

Mercy N. Ndalila[1], Grant J. Williamson[1], and David M. J. S. Bowman[1]

Additional File 2

Table S1: Comparison of total emissions (in Tg) and per-hectare emissions (in t ha^{-1}) among wildfires in Australia. Burnt area estimates (BA; in ha) for each fire event are indicated in rounded brackets. CO_2-equivalent (CO_2-e) emissions are totals from CO_2, methane and nitrous oxide emissions. The estimate for the Forcett-Dunalley fire (this study) has also been compared with estimates from the FullCAM model that is used in Australia for national GHG accounting.

Fire event (BA)	**CO_2 emissions**		**$PM_{2.5}$ emissions**	
	Emission (Tg)	Area-normalized (t ha^{-1})	Emission (Tg)	Area-normalised (t ha^{-1})
**Australia**				
This study (20,200 ha)	1.125	55.7<br>142 (*FullCAM*)	0.022 ± 0.006	1.08
2003 Canberra forest fire [1] (260,000)	20.2	78	-	-
2009 Black Saturday fires, Victoria [2] (267,200 -GFED)	18	67.4	-	-
2019-2020 Eastern Australian *Euc.* Fires [1] (7.4 M)	850<br>940 CO_2-e	115<br>130 CO_2-e	-	-
2013 *Euc.* forest fire, Victoria [3]	-	105 -long-unburnt<br>42 -fuel-reduced		
Four 2015 prescribed fires in Victorian forests [4] (56-293)	-	-	-	0.073–0.164
Regeneration fire in southern Tasmanian native forest [5] (1,127)	-	-	0.008	6.91
2000-2004 Western Arnhem savanna fires [6] (2,389,300)	0.272 CO_2-e p.a.	0.11	-	-

References

1. Commonwealth of Australia: **Estimating greenhouse gas emissions from bushfires in Australia's temperate forests: focus on 2019-20**: Department of Industry, Science, Energy and Resources; 2020.
2. Paton-Walsh C, Emmons LK, Wiedinmyer C: **Australia's Black Saturday fires – Comparison of techniques for estimating emissions from vegetation fires**. *Atmos Environ* 2012, **60**:262-270.

3. Volkova L, Meyer CM, Murphy S, Fairman T, Reisen F, Weston C: **Fuel reduction burning mitigates wildfire effects on forest carbon and greenhouse gas emission**. *Int J Wildland Fire* 2014, **23**(6):771-780.
4. Reisen F, Meyer CP, Weston CJ, Volkova L: **Ground-Based Field Measurements of PM2.5 Emission Factors From Flaming and Smoldering Combustion in Eucalypt Forests**. *J Geophys Res Atmos* 2018, **123**(15):8301-8314.
5. Meyer CP, Reisen F, Keywood MD, Crumeyrolle S: **Impacts of smoke from regeneration burning on air quality in the Huon Valley, Tasmania**. CSIRO, Melbourne, Australia; 2011.
6. Russell-Smith J, Murphy BP, Meyer CP, Cook GD, Maier S, Edwards AC, Schatz J, Brocklehurst P: **Improving estimates of savanna burning emissions for greenhouse accounting in northern Australia: limitations, challenges, applications**. *Int J Wildland Fire* 2009, **18**(1):1-18.
